# The "Mucous Disease" of Children

**Published:** 1912-09-21

**Authors:** 


### THE "MUCOUS DISEASE" OF CHILDREN.

### A Summary of Symptoms and Treatment.

This disorder is of frequent occurrence, yet is often overlooked because one or other of its symptoms happens, by dominating the rest, to distort the clinical picture and lead the observer astray.

Whether the condition should be deemed a distinct disease or not, it is certainly desirable that it should be separated from the disorderly chaos called "indigestion"; and the name "mucous disease," while not covering the whole condition by any means, at least indicates its predominant feature, for it is a chronic morbid condition in which all the mucous membranes are apt to be affected in varying degrees by hypersecretion of mucus.

The mucous membrane of the alimentary canal is most affected, but generally evidence can be obtained that the naso-pharynx, the bronchi, and perhaps the urinary tract are also unhealthy.

### General Symptoms.

The condition is perhaps a neurosis (comparable to the mucous colitis of women). It occurs in children about the beginning of the second dentition, and more often in boys than girls. The parents observe a persistent and apparently inexplicable poverty of appetite accompanied by loss of weight, or at least by a failure to increase in weight at the proper rate. Add to this, pallor of the face and general lassitude and a constant dry little cough, and it becomes easy to understand the parents' fear lest their child be falling into consumption. Sometimes the appetite, on the contrary, is "ravenous," and the child will eat strange things such as coal, earth, and the like.

On inspection one notes a pale thin child, often with dark rings round the eyes. The tongue is dry, either smooth and glazed or thinly coated with white. The breath is offensive.

The tonsils are large and flabby; the posterior pharyngeal wall is dry and glazed with sticky secretion adherent to it; and often there are adenoids as well; the state of the naso-pharynx is sufficient to explain the cough from which such a patient may suffer.

On examination of the abdomen (the skin of which is dry and harsh) one observes it is swollen so as to suggest at first sight tuberculosis of the peritoneum, but on palpation nothing abnormal is discovered unless it be distension of intestine with flatus.

There is history of curious waves of pallor appearing suddenly in the face for a few moments and then disappearing, so that the child is said "to turn faint very easily." In addition, the child is subject to sharp attacks of colicky pain in the abdomen, irrespective of meal times; the action of the bowels is irregular. Sometimes diarrhœa, especially immediately after meals, is a troublesome and persistent feature; here the stools are loose, pale, and offensive; and this may alternate with fits of obstinate constipation when the scybalus masses are enmeshed in mucus, often enough accompanied by threadworms.

Given, in addition, an occasional rise of temperature, and it is no wonder that the observer thinks of "catarrhal appendicitis"; but in reality the morbid condition is not limited to the appendix, and so operation is an unnecessary and useless burden to inflict on the patient. There will sometimes be attacks of vomiting lasting perhaps a day or two, without any obvious cause to explain them. The urine is apt to be loaded with urates and may easily show a trace of albumen towards the end of the day. Lastly, the nervous system is disturbed, and we are told of such symptoms as night terrors, teeth-grinding in the sleep, enuresis, and the like.

In treating these cases attention to diet is of paramount importance, for without that even the most elaborate prescription will fail to effect a lasting improvement. Accordingly we cut off sugar (especially sweets and cooked sugar), and reduce to a minimum the starch foods—potatoes, bread, and porridge.

One observes that the patients are often the children of parents with unwholesome nervous systems, a point which is in keeping with the idea that the disease is based upon a neurosis. This view is supported by the vaso-motor spasms producing the waves of pallor, by the intestinal spasms not wholly to be explained by the mechanical presence of mucus, by the night terrors and enuresis; and it is significant that the disease is apt to show itself at the age when a child begins to go to school. Moreover, some cases are cured simply by a good holiday. However this may be, we have generally to use drugs in dealing with the more pronounced symptoms.

It is generally wise to give aperients; and of these mag. sulph. with alkalies seems most satisfactory. When diarrhœa is a troublesome feature hyd. c. creta and possibly opium is frequently recommended, although I have myself found the pancreatic ferments most serviceable. Rhubarb is a favourite drug with some, but the cases which a change of diet alone fail to cure generally require something more specific; from the condition of the stools there seems to be a deficiency of pancreatic secretion, and on this ground it is reasonable to employ preparations of pancreatic ferment in preference to ordinary drugs.

Lastly, we may need, in bad cases, to employ more direct means for the removal of mucus from stomach and colon; a few days of stomach washing and colon irrigation serve to give the patient a fresh start, and reduce the hypersecretion of mucus to proportions more amenable to drugs.

In children of seven and upwards, one must not ignore the question of school and overwork. It is these who benefit by a judicious holiday.

